# Effects of Acute Beetroot Juice Ingestion and Hypoxic Conditions on Metabolic Function, Skeletal Muscle Oxygenation, and Cardiac Function During Endurance Exercise in Healthy Young Men

**DOI:** 10.3390/nu18172780

**Published:** 2026-08-25

**Authors:** Jae-Ho Choi, Eunjoo Lee, Hun-Young Park, Won-Sang Jung, Seungyeon Woo, Su young Kim, Yuanning Zheng, Seong Hyun Park, Yerin Sun, Sung-Woo Kim

**Affiliations:** 1 Department of Sports Medicine and Science, Graduate School, Konkuk University, Seoul 05029, Republic of Korea; zas1135@konkuk.ac.kr (J.-H.C.); eunjooo@konkuk.ac.kr (E.L.); parkhy1980@konkuk.ac.kr (H.-Y.P.); wsyzz92@konkuk.ac.kr (S.W.); syk6919@konkuk.ac.kr (S.y.K.); qw1204153841@konkuk.ac.kr (Y.Z.); pjin252@konkuk.ac.kr (S.H.P.); 2 Physical Activity and Performance Institute, Konkuk University, Seoul 05029, Republic of Korea; 3 Department of Senior Exercise Prescription, Dongseo University, 47, Jurye-ro, Sasang-gu, Busan 47011, Republic of Korea; jws1197@dongseo.ac.kr; 4 Department of Gerontology, AgeTech-Service Convergence Major, Graduate School of East-West Medical Science, Kyung Hee University, Yongin 17104, Republic of Korea; yleen06@gmail.com

**Keywords:** hypoxic condition, normoxic condition, beetroot juice, nitric oxide, skeletal muscle oxygenation, metabolic responses, cardiac function

## Abstract

Background/Objectives: Exercise in hypoxic environments induces greater physiological stress than normoxic conditions, potentially limiting exercise performance through reduced skeletal muscle oxygenation and increased metabolic strain. Beetroot juice (BRJ), a dietary source of inorganic nitrate, may enhance nitric oxide (NO) bioavailability and oxygen delivery under hypoxic conditions. This study examined the acute effects of BRJ supplementation on metabolic responses, skeletal muscle oxygenation, and cardiac function during submaximal exercise at a fixed heart rate under normoxic and hypoxic conditions. Methods: Twelve healthy adult males completed four conditions in a randomized, crossover design, with double-blinding applied to the supplementation condition: normoxic condition + placebo (NPLA), normoxic condition + BRJ (NBRJ), hypoxic condition + placebo (HPLA), and hypoxic condition + BRJ (HBRJ). Participants ingested 70 mL of BRJ (400 mg NO_3_^−^) or placebo 2.5 h before 30 min of submaximal exercise at 70% HRmax under normoxic (FiO_2_ 20.9%) or hypoxic (FiO_2_ 14.5%) conditions. A two-way repeated-measures ANOVA was used to test the main effects of environment and treatment and their interaction for each outcome. Results: Hypoxic conditions required a significantly lower mechanical load and induced greater physiological stress. However, a significant environment × treatment interaction was observed for Deoxy_Hb, with HBRJ statistically indistinguishable from normoxic conditions, indicating a clear attenuation of the hypoxia-induced increase in oxygen extraction. In addition, a significant main effect of treatment without a significant interaction was observed for RER, and HBRJ did not differ significantly from NBRJ. Conclusions: BRJ supplementation may help attenuate hypoxia-induced skeletal muscle deoxygenation, as reflected in Deoxy_Hb, potentially via NO-mediated mechanisms; however, as circulating nitrate/nitrite concentrations were not directly measured and interaction effects were not confirmed for most variables, these findings should be regarded as preliminary evidence for the potential use of BRJ as a nutritional strategy in hypoxic exercise settings.

## 1. Introduction

Exercise in hypoxic environments has emerged as an effective non-pharmacological intervention strategy not only for enhancing athletic performance in elite athletes but also for promoting health in the general population and individuals at risk of obesity and metabolic diseases [1,2,3,4]. Hypoxic environments, whether through natural high-altitude exposure or normobaric hypoxia induced in controlled laboratory settings, reduce the fraction of inspired oxygen (FiO_2_), limiting the amount of oxygen delivered to tissues, and are known to induce greater physiological and metabolic stimulation than normoxic environments even at the same relative exercise intensity [5,6]. These hypoxic stimuli activate various hypoxia-adaptive mechanisms, including hypoxia-inducible factor-1α (HIF-1α), vascular endothelial growth factor (VEGF), and glucose transporter type 4 (GLUT-4), thereby enhancing vascular and metabolic function, and have consequently been applied as an effective exercise intervention strategy for improving exercise performance and promoting cardiometabolic health [7,8,9,10].

However, in hypoxic environments, the limited oxygen supply during exercise can lead to decreased skeletal muscle oxygenation and increased fatigue accumulation, potentially impairing exercise performance [11]. In particular, even at the same exercise intensity, hypoxia may elicit increased minute ventilation, greater cardiovascular strain, and reduced oxygen delivery efficiency, all of which may act as limiting factors for the beneficial effects of hypoxic exercise [11]. Therefore, there is growing interest in nutritional intervention strategies that can further enhance oxygen delivery and utilization efficiency to peripheral tissues while preserving the physiological benefits of hypoxic exercise.

Among such strategies, beetroot juice (BRJ), a rich source of dietary inorganic nitrate, has received increasing attention. BRJ contains a variety of bioactive compounds, including inorganic nitrate (NO_3_^−^), betalains, and polyphenols, and is recognized as a representative dietary source that increases nitric oxide (NO) bioavailability through the NO_3_^−^–nitrite (NO_2_^−^)–NO pathway [12,13,14]. Upon ingestion, dietary NO_3_^−^ is reduced to NO_2_^−^ and subsequently to NO, and this process is further facilitated under hypoxic and acidic conditions [15,16]. The resultant NO has been reported to contribute to improvements in exercise economy and exercise performance through vasodilation, increased blood flow, and enhanced endothelial function, as well as improvements in skeletal muscle oxygen utilization efficiency and mitochondrial function [12,14,17].

However, existing research on the combined effects of BRJ supplementation and hypoxic exposure has largely focused on outcome measures assessed before and after exercise, such as maximal oxygen uptake (VO_2_max), time trial performance, maximal strength, and exercise performance, and studies that have comprehensively evaluated metabolic responses, skeletal muscle oxygenation, and cardiac function during exercise are extremely limited [16,18,19]. In addition, several studies have reported that BRJ supplementation does not elicit an ergogenic effect under hypoxic conditions, suggesting that this effect is not consistently observed across all contexts [20,21]. This inconsistency may reflect the fact that the physiological mechanisms underlying the combined effects of BRJ and hypoxic exposure have not yet been clearly elucidated, and the limited number of studies comprehensively evaluating real-time physiological responses during exercise represents a gap that has not been sufficiently addressed in this regard. Therefore, this study aimed to examine the effects of BRJ supplementation on metabolic responses, skeletal muscle oxygenation, and cardiac function during exercise under normoxic and hypoxic conditions, thereby elucidating the physiological mechanisms underlying the combined effects of BRJ and hypoxic exposure.

## 2. Materials and Methods

### 2.1. Participants

The participants in this study consisted of twelve healthy adult males with normal body weight who were non-smokers and had no history of musculoskeletal, metabolic, or pulmonary disease. All participants’ health status was confirmed using the 2020 Physical Activity Readiness Questionnaire for Everyone (PAR-Q+), and each participant voluntarily provided written informed consent after receiving a thorough explanation of the purpose and procedures of the study.

The sample size for this study was calculated based on the effect size (partial η^2^ = 0.647) reported for the treatment (supplementation) effect on VCO_2_, derived from repeated-measures ANOVA in a previous study [22]. Using G*Power (version 3.1.9.7) with a significance level (α) of 0.05 and statistical power (1 − β) of 0.90, the minimum required sample size was calculated to be 10 participants. However, considering the possibility of dropout during the study, a total of 12 participants were ultimately recruited.

The physical characteristics of the participants are presented in Table 1, and peripheral oxygen saturation (SpO_2_) during exercise under each experimental condition is presented in Table 2. This study was approved by the Institutional Review Board (IRB) of Konkuk University (Approval No. 7001355-202208-HR-573), and all study procedures were conducted in accordance with the ethical principles of the Declaration of Helsinki. This trial was registered at the Clinical Research Information Service (CRIS) (Registration No. KCT0012010).

### 2.2. Study Design

All participants visited the laboratory a total of five times during the experimental period. At the first visit, after a minimum of 4 h of fasting, height and body composition were measured, followed by a familiarization trial to allow participants to become accustomed to the exercise protocol used in the main experiment. The familiarization trial consisted of 30 min of cycle ergometer exercise performed at a target heart rate (HR) corresponding to 70% of maximal HR (HRmax), which was calculated using the Miyashita equation (male = 209 − 0.69 × age), based on a protocol modified from a previous study [11].

**Figure 1:**
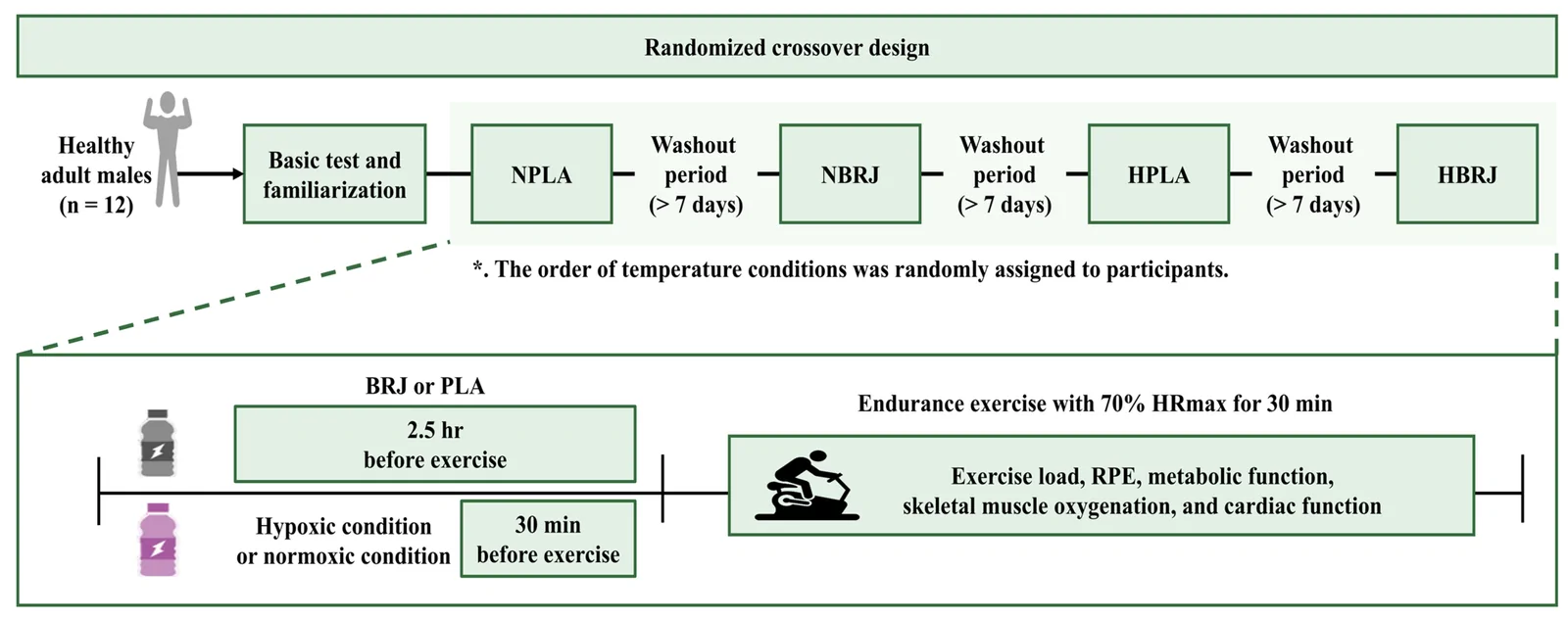
Study design. NPLA, normoxic condition+ placebo; NBRJ, normoxic condition + beetroot juice; HPLA, hypoxic condition + placebo; HBRJ, hypoxic condition + beetroot juice; BRJ, beetroot juice; PLA, placebo; FiO_2_, fraction of inspired oxygen; HRmax, maximal heart rate; HR, heart rate; RPE, rating of perceived exertion; SpO_2_, peripheral oxygen saturation; VE, minute ventilation; VO_2_, oxygen uptake; VCO_2_, carbon dioxide production; RER, respiratory exchange ratio; O_2_pulse, oxygen pulse; Oxy_Hb, oxygenated hemoglobin; Deoxy_Hb, deoxygenated hemoglobin; Total_Hb, total hemoglobin; StO_2_, tissue oxygen saturation; SV, stroke volume; CO, cardiac output.

From the second to the fifth visits, participants completed all four conditions in a randomized crossover design: normoxic condition + placebo (NPLA), normoxic condition + beetroot juice (NBRJ), hypoxic condition + placebo (HPLA), and hypoxic condition + beetroot juice (HBRJ) (Figure 1). All experimental sessions for a given participant were conducted at the same time of day to minimize potential circadian influences on the measured outcomes. The order of the four conditions was determined using a random number table, and a Latin square design was applied to ensure that each condition appeared equally across all visit positions, achieving full counterbalancing. The treatment order was assigned by a researcher not involved in data collection so that the researchers conducting the measurements remained unaware of the upcoming condition. Because the normoxic and hypoxic conditions are directly perceptible to participants, double-blinding could not be applied to the environmental factor; however, double-blinding was maintained for the supplementation condition (BRJ vs. PLA).

A washout period of 7 days was implemented between each condition, based on durations established in the previous literature, to minimize the carryover effects of the previous treatment. Prior to each visit, participants were instructed to refrain from using mouthwash and to limit their intake of nitrate-rich cruciferous vegetables (e.g., broccoli, cauliflower, and red cabbage) [23]. In addition, all physical activity was restricted for 24 h before each experimental trial, and participants were prohibited from brushing their teeth on the day of the experiment to preserve oral nitrate-reducing bacteria [23,24]. Compliance with these instructions was verbally confirmed with each participant at every visit.

All participants ingested either PLA or BRJ 2.5 h prior to the start of exercise. Following ingestion, participants underwent a 30 min acclimatization period under either normoxic conditions (FiO_2_: 20.9%) or hypoxic conditions (FiO_2_: 14.5%), after which they performed 30 min of endurance exercise at the same heart rate level (70% of HRmax). During the 30 min endurance exercise, exercise load, rating of perceived exertion (RPE), metabolic function, skeletal muscle oxygenation, and cardiac function were measured.

### 2.3. Exercise Protocol

To compare physiological responses between hypoxic and normoxic conditions at an equivalent internal load independent of external workload, exercise intensity was fixed at 70% HRmax [11] using a cycle ergometer (Aerobike 75XLII; Konami Corporation, Tokyo, Japan), which automatically adjusts power output (watts) in real time to maintain the pre-set target heart rate. This HR-based approach standardizes relative exercise intensity across participants with differing fitness levels and isolates the physiological effect of hypoxia from that of external workload, consistent with common practice in hypoxic exercise research [25]. The exercise program consisted of 30 min of submaximal endurance exercise, including the time required to reach the target heart rate established during the familiarization trial.

### 2.4. Supplementation

**Figure 2:**
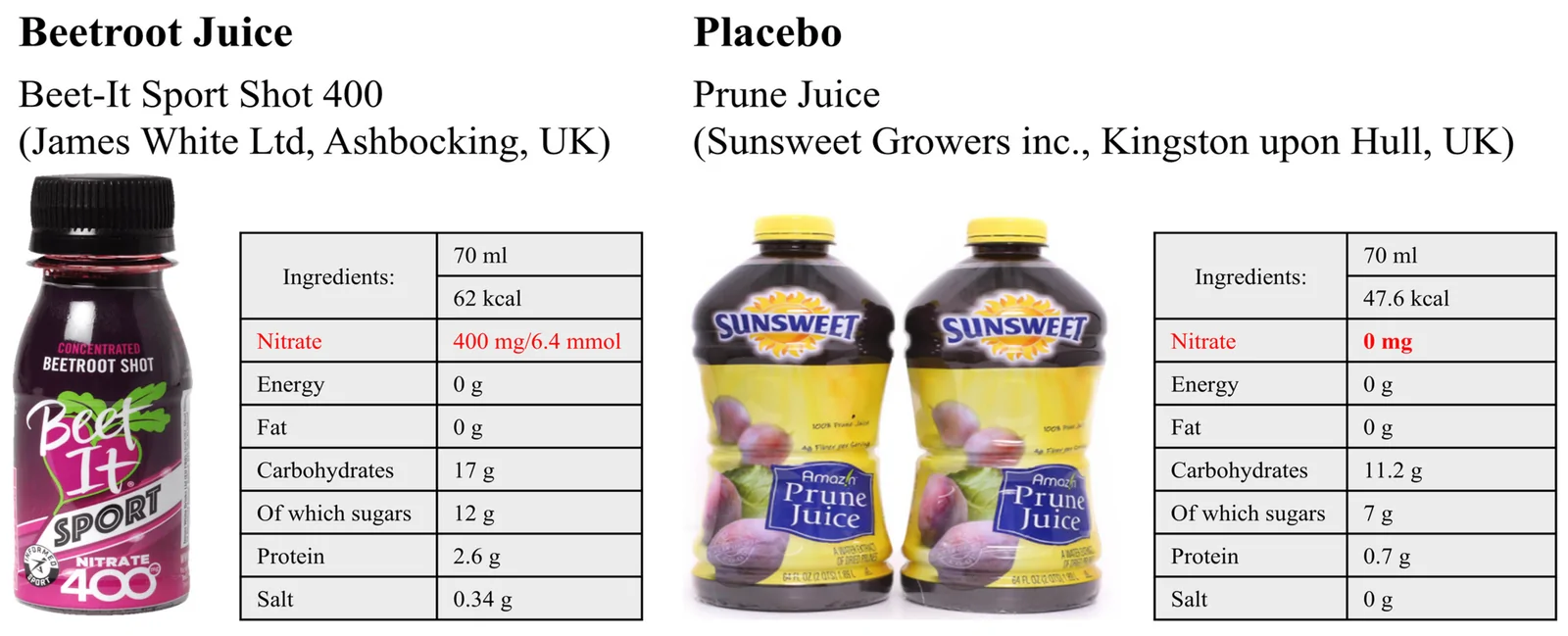
Beetroot Juice and Placebo Supplementation.

BRJ was provided as 70 mL of Beet-It Sport Shot 400 (James White Ltd., Ipswich, UK), containing 400 mg of dietary nitrate (NO_3_^−^). PLA consisted of prune juice (Sunsweet Growers Inc., Kingston upon Hull, UK), which is similar in color and viscosity to BRJ and contains a negligible amount of dietary nitrate (<0.01 mM) (Figure 2). The differences in carbohydrate and dietary fiber content between BRJ and PLA have been reported to have no effect on physiological responses [26]. All participants ingested either BRJ or PLA 2.5 h prior to the start of exercise. Participants were informed in advance that temporary, red-colored urine or stools may occur following BRJ ingestion [27]. No adverse events related to supplement ingestion were observed in this study.

### 2.5. Outcome Measurements

#### 2.5.1. Height and Body Composition

Height and body composition were measured in the morning after maintaining a fast (no food or water intake) for at least 4 h, using an automatic stadiometer (BSM 330, InBody, Seoul, Republic of Korea) and a body composition analyzer (InBody 770, InBody, Seoul, Republic of Korea). The measured variables included height (cm), body weight (kg), body mass index (BMI, kg/m^2^), fat-free mass (kg), body fat mass (kg), and percent body fat (%).

#### 2.5.2. Exercise Load and Rating of Perceived Exertion

Exercise load was continuously recorded at 1 min intervals during the 30 min submaximal endurance exercise performed at 70% of HRmax, using a cycle ergometer. The measured variable was exercise load (watts). RPE was assessed at 1 min intervals using the Borg 6–20 scale, and data were subsequently averaged into consecutive 5 min intervals (i.e., 0–5, 5–10, 10–15, 15–20, 20–25, and 25–30 min) for analysis.

#### 2.5.3. Metabolic Response

Metabolic responses were continuously recorded at 1 min intervals during the 30 min submaximal endurance exercise performed at 70% of HRmax, using an automated breath-by-breath metabolic analyzer (K5, Cosmed, Rome, Italy) with a facemask-type respiratory valve. The measured variables included minute ventilation (VE), oxygen uptake (VO_2_), carbon dioxide excretion (VCO_2_), respiratory exchange ratio (RER), and oxygen pulse (O_2_pulse). O_2_pulse was calculated by dividing VO_2_ by heart rate (HR) at each time point, while the remaining variables were automatically measured by the device.

#### 2.5.4. Skeletal Muscle Oxygenation

Skeletal muscle oxygenation was continuously recorded at 1 min intervals at the vastus lateralis during the 30 min submaximal endurance exercise performed at 70% of HRmax, using a near-infrared spectroscopy (NIRS) system (Astem, Kawasaki, Japan). The measured variables included oxygenated hemoglobin (oxy_Hb), deoxygenated hemoglobin (deoxy_Hb), total hemoglobin (Total_Hb), and tissue oxygen saturation (StO_2_).

#### 2.5.5. Cardiac Function

Cardiac function was continuously recorded at 1 min intervals during the 30 min submaximal endurance exercise performed at 70% of HRmax, using a thoracic bioelectrical impedance device (PhysioFlow PF-05; Manatec Biomedical, Paris, France), which consisted of two impedance electrocardiogram electrodes placed over the supraclavicular fossa at the base of the left side of the neck, two electrocardiogram electrodes used for ECG recording, and two electrodes placed at the xiphoid process. The measured variables included heart rate (HR), stroke volume (SV), and cardiac output (CO).

### 2.6. Statistical Analysis

All statistical analyses were performed using SPSS version 30.0 (IBM Corp., Armonk, NY, USA). All data are presented as mean ± standard deviation (SD). Prior to parametric statistical analysis, the normality of all dependent variables was confirmed using the Shapiro–Wilk test.

A two-way repeated-measures ANOVA, with environment and supplementation as within-subject factors, was conducted for each outcome variable to test the main effect of environment, the main effect of supplementation, and the environment × treatment interaction, with the area under the curve (AUC) used for exercise load and metabolic responses, and the mean value used for skeletal muscle oxygenation and cardiac function, both calculated over the entire 30 min exercise period. When a significant interaction was observed, a one-way repeated-measures ANOVA across the four conditions followed by Bonferroni post hoc tests was additionally performed to further characterize the nature of the interaction. When a significant main effect was observed in the absence of a significant interaction, the effect of supplementation within each environment and the effect of environment within each supplementation condition were compared using paired *t*-tests. Mauchly’s test was used to assess the assumption of sphericity for each dependent variable, and when this assumption was violated, the Greenhouse–Geisser correction was applied. Effect sizes are reported as partial eta-squared (*η_p_*^2^) for all ANOVA comparisons. A two-way repeated-measures ANOVA with condition × time as within-subject factors was conducted to examine the interaction and main effects on RPE; when a significant interaction or main effect was identified, Bonferroni post hoc tests were performed to determine differences between conditions. Statistical significance was set at *p* < 0.05.

## 3. Results

### 3.1. Exercise Load and Rating of Perceived Exertion

**Figure 3:**
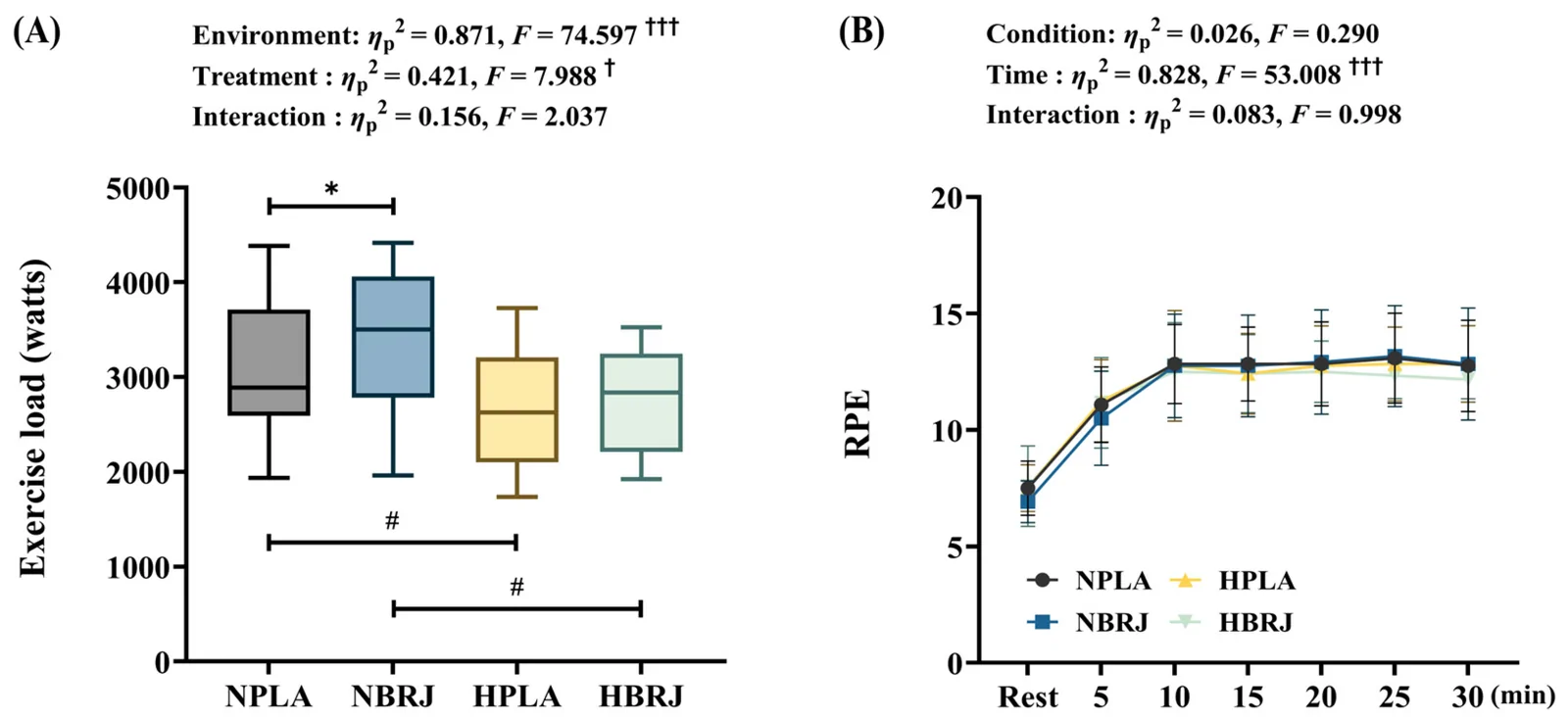
Exercise load and RPE during 30 min of submaximal endurance exercise at 70% HRmax. (**A**) Exercise load; (**B**) RPE. NPLA, normoxic condition + placebo; NBRJ, normoxic condition + beetroot juice; HPLA, hypoxic condition + placebo; HBRJ, hypoxic condition + beetroot juice; RPE, rating of perceived exertion. ^†^ *p* < 0.05, ^†††^ *p* < 0.001, significant main effect of condition or time. * Indicates a significant difference between PLA and BRJ within the same environment (*p* < 0.05). # Indicates a significant difference between normoxic and hypoxic conditions within the same supplementation condition (*p* < 0.05).

Figure 3 presents the exercise load and RPE during the 30 min submaximal endurance exercise across the four conditions. A significant main effect of environment (*F* = 74.597, *p* < 0.001) and supplementation (*F* = 7.988, *p* = 0.016) was observed for exercise load, whereas the interaction was not significant (*F* = 2.037, *p* = 0.181). Post hoc analysis revealed that exercise load was significantly higher in NBRJ than in NPLA, and both hypoxic conditions (HPLA and HBRJ) showed significantly lower exercise loads compared to both normoxic conditions (NPLA and NBRJ). No significant differences were observed between HPLA and HBRJ.

For RPE, no significant main effect of condition or interaction effect was observed.

### 3.2. Metabolic Responses

**Figure 4:**
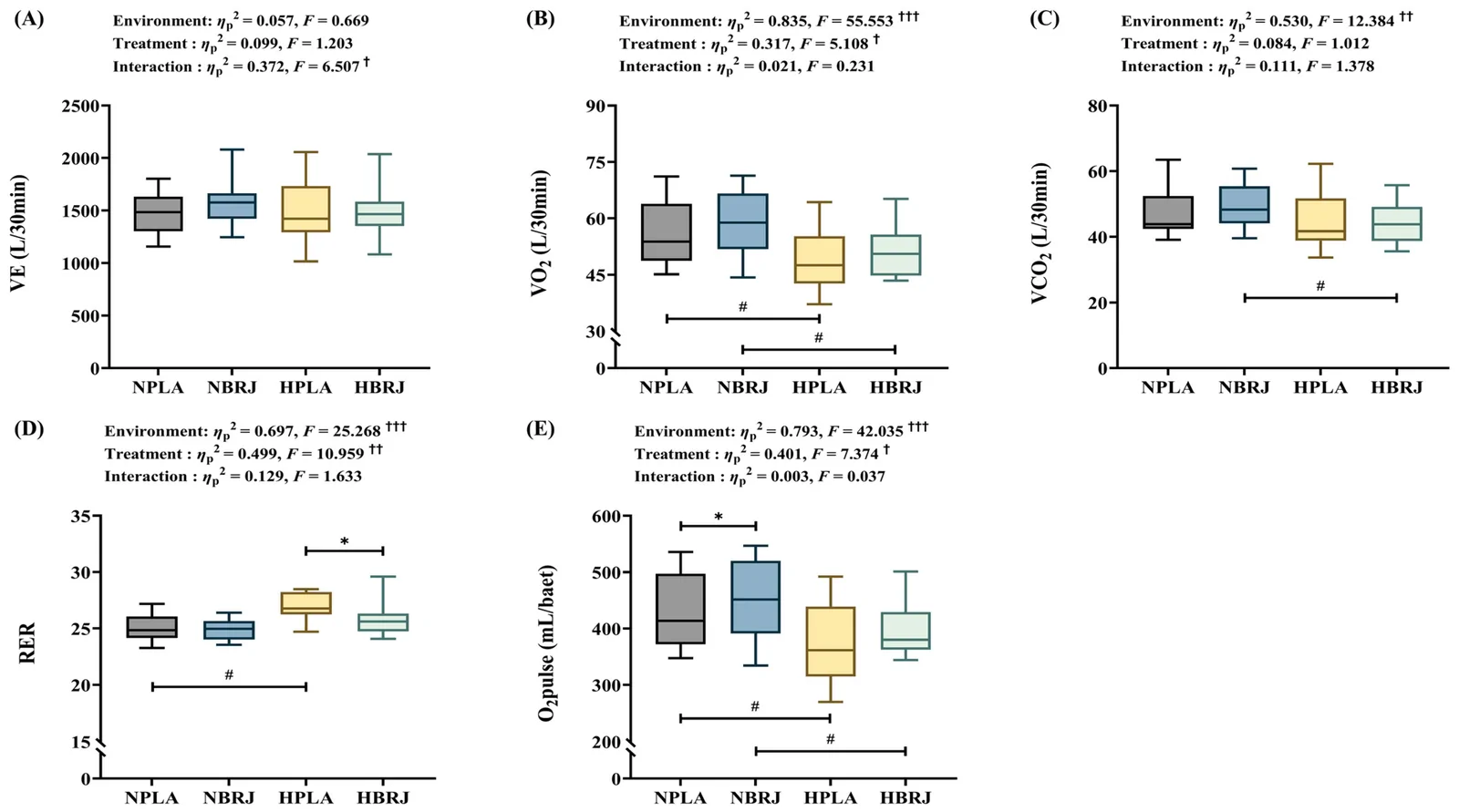
Metabolic responses during 30 min of submaximal endurance exercise at 70% HRmax. (**A**) VE; (**B**) VO_2_; (**C**) VCO_2_; (**D**) RER; (**E**) O_2_pulse. NPLA, normoxic condition + placebo; NBRJ, normoxic condition + beetroot juice; HPLA, hypoxic condition + placebo; HBRJ, hypoxic condition + beetroot juice; VE, minute ventilation; VO_2_, oxygen uptake; VCO_2_, carbon dioxide excretion; RER, respiratory exchange ratio; O_2_pulse, oxygen pulse. ^†^ *p* < 0.05, ^††^ *p* < 0.01, ^†††^ *p* < 0.001, significant main effect of condition. * Indicates a significant difference between PLA and BRJ within the same environment (*p* < 0.05). # Indicates a significant difference between normoxic and hypoxic conditions within the same supplementation condition (*p* < 0.05).

Figure 4 presents metabolic responses during the 30 min submaximal endurance exercise across the four treatments. A significant environment × treatment interaction was observed for VE (*F* = 6.507, *p* = 0.027). However, post hoc analysis revealed no significant differences among the four conditions.

For VO_2_, significant main effects of environment (*F* = 55.553, *p* < 0.001) and supplementation (*F* = 5.108, *p* = 0.045) were observed. Post hoc analysis revealed that VO_2_ was significantly lower in both hypoxic conditions (HPLA and HBRJ) compared to both normoxic conditions (NPLA and NBRJ).

For VCO_2_, a significant main effect of environment was observed (*F* = 12.384, *p* = 0.005), whereas no significant main effect of supplementation was found. Post hoc analysis revealed that VCO_2_ was significantly higher in NBRJ than in HBRJ, whereas no significant difference was observed between NPLA and HPLA.

For RER, significant main effects of environment (*F* = 25.268, *p* < 0.001) and supplementation (*F* = 10.959, *p* = 0.007) were observed. Post hoc analysis revealed that RER was significantly higher in HPLA than in both NPLA and HBRJ. However, no significant difference was observed between HBRJ and NBRJ.

For O_2_pulse, significant main effects of environment (*F* = 42.035, *p* < 0.001) and supplementation (*F* = 7.374, *p* = 0.020) were observed. Post hoc analysis revealed that O_2_pulse was significantly higher in NBRJ than in NPLA and that both hypoxic conditions (HPLA and HBRJ) were significantly lower than both normoxic conditions (NPLA and NBRJ).

### 3.3. Skeletal Muscle Oxygenation

**Figure 5:**
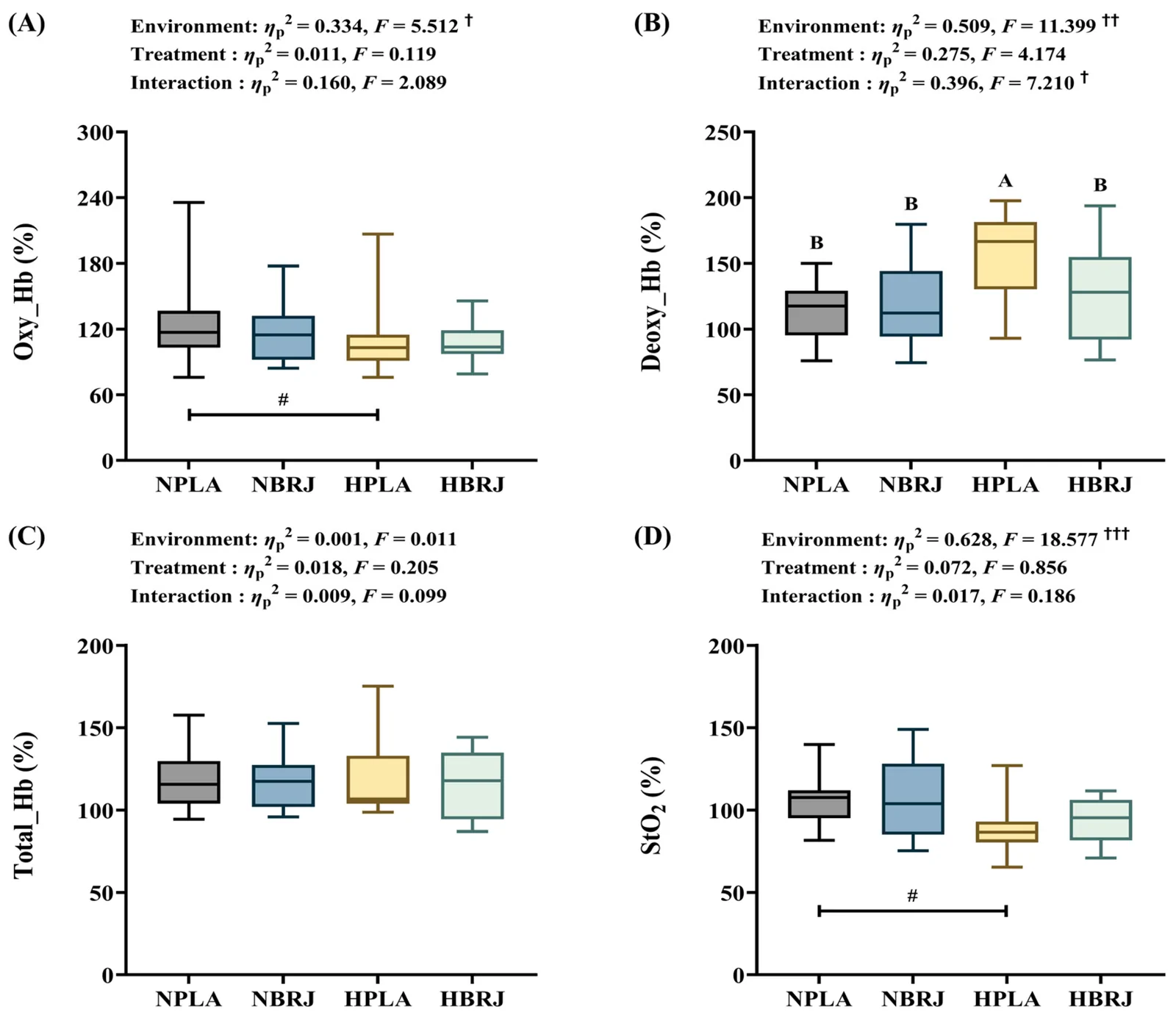
Skeletal muscle oxygenation during 30 min of submaximal endurance exercise at 70% HRmax. (**A**) Oxy_Hb; (**B**) Deoxy_Hb; (**C**) Total_Hb; (**D**) StO_2_. NPLA, normoxic condition + placebo; NBRJ, normoxic condition + beetroot juice; HPLA, hypoxic condition + placebo; HBRJ, hypoxic condition + beetroot juice; Oxy_Hb, oxygenated hemoglobin; Deoxy_Hb, deoxygenated hemoglobin; Total_Hb, total hemoglobin; StO_2_, tissue oxygen saturation. ^†^ *p* < 0.05, ^††^ *p* < 0.01, ^†††^ *p* < 0.001, significant main effect of condition. Different letters (A, B) indicate significant differences between conditions (*p* < 0.05). # Indicates a significant difference between normoxic and hypoxic conditions within the same supplementation condition (*p* < 0.05).

Figure 5 presents skeletal muscle oxygenation during the 30 min submaximal endurance exercise across the four treatments. A significant environment × treatment interaction was observed for Deoxy_Hb (*F* = 7.210, *p* = 0.021). Post hoc analysis revealed that Deoxy_Hb was significantly higher in HPLA than in NPLA, NBRJ, and HBRJ.

Significant main effects of environment were observed for Oxy_Hb (*F* = 5.512, *p* = 0.039) and StO_2_ (*F* = 18.577, *p* = 0.001). Post hoc analysis revealed that Oxy_Hb and StO_2_ were significantly lower in HPLA than in NPLA.

No significant main or interaction effects were observed for Total_Hb.

### 3.4. Cardiac Function

**Figure 6:**
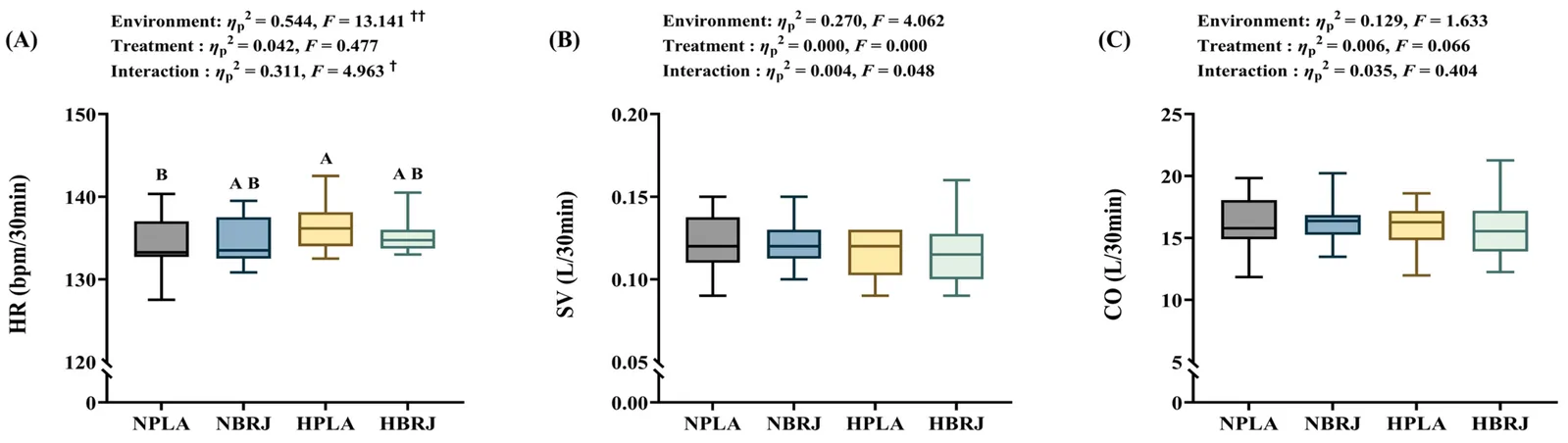
Cardiac function during 30 min of submaximal endurance exercise at 70% HRmax. (**A**) HR; (**B**) SV; (**C**) CO. NPLA, normoxic condition + placebo; NBRJ, normoxic condition + beetroot juice; HPLA, hypoxic condition + placebo; HBRJ, hypoxic condition + beetroot juice; HR, heart rate; SV, stroke volume; CO, cardiac output. ^†^ *p* < 0.05, ^††^ *p* < 0.01, significant main effect of condition. Different letters (A, B) indicate significant differences between conditions (*p* < 0.05).

Figure 6 presents cardiac function during the 30 min submaximal endurance exercise across the four treatments. A significant environment × treatment interaction was observed for HR (*F* = 4.963, *p* = 0.048). Post hoc analysis revealed that HR was significantly higher in HPLA than in NPLA.

However, no significant main or interaction effects were observed for SV or CO.

## 4. Discussion

The present study compared the effects of BRJ supplementation on metabolic responses, skeletal muscle oxygenation, and cardiac function during exercise at the same heart rate level (70% HRmax) under normoxic and hypoxic conditions. The primary findings indicated that the mechanical load required to maintain the same heart rate level was significantly lower under hypoxic conditions than under normoxic conditions and that BRJ supplementation resulted in a significantly higher mechanical load than placebo under normoxic conditions. RPE remained similar across all four conditions. Notably, Deoxy_Hb was higher in HPLA than in NPLA, NBRJ, and HBRJ, and HR was higher in HPLA than in NPLA, although HBRJ did not differ significantly from the normoxic condition. RER was significantly higher in HPLA than in both NPLA and HBRJ, and O_2_pulse was significantly higher in NBRJ than in NPLA.

In the present study, hypoxic conditions (HPLA and HBRJ) exhibited significantly lower mechanical load compared to normoxic conditions (NPLA and NBRJ). This is likely because the reduction in FiO_2_ under hypoxic conditions decreases arterial oxygen saturation, which stimulates peripheral chemoreceptors and increases sympathetic nervous system activity, leading to an accelerated increase in HR [28], thereby requiring a lower mechanical load than normoxic conditions to maintain the same target HR level [11,25]. These findings are consistent with previous studies reporting that hypoxic conditions induce lower mechanical load than normoxic conditions at the same HR level [11,25]. On the other hand, NBRJ showed a significantly higher mechanical load than NPLA. This may be attributed to activation of the NO_3_^−^–NO_2_^−^–NO pathway through BRJ supplementation, which enhances oxygen delivery efficiency to skeletal muscle via vasodilation and increased blood flow, thereby improving mitochondrial oxidative phosphorylation efficiency and exercise economy. These adaptations may have attenuated the reduction in phosphocreatine (PCr) and the accumulation of adenosine diphosphate (ADP) and inorganic phosphate (Pi) during exercise, enabling a higher mechanical load to be sustained at the same heart rate level [12,14,17]. This is consistent with a previous study using a similar protocol (70% HRmax), which reported that BRJ supplementation significantly increased exercise load and improved flow-mediated dilation (FMD) and pulse wave velocity (PWV) [14]. However, since circulating nitrate/nitrite concentrations and intramuscular PCr, ADP, and Pi levels were not directly measured in the present study, this mechanism represents a hypothetical interpretation based on prior literature, and caution is warranted in its interpretation. In contrast, HBRJ did not differ significantly from HPLA in mechanical load. Since the present study employed a same heart rate HR design in which exercise intensity was continuously adjusted to maintain the target HR, the increased physiological stress induced by hypoxic conditions was reflected as a reduction in mechanical load required to achieve the target HR [11,25]. Therefore, even if BRJ supplementation induced NO-mediated vasodilation and improved blood flow, as suggested above [14], these potential effects may not have been sufficient to induce an additional meaningful change in the mechanical load required to reach the target HR. Furthermore, it cannot be ruled out that hypoxic conditions acted as a potent physiological stimulus that markedly reduced mechanical load, thereby limiting the relative contribution of BRJ supplementation. Despite these differences in mechanical load across conditions, RPE did not differ significantly among NPLA, NBRJ, HPLA, and HBRJ. This is likely because all participants performed exercise at the same relative exercise intensity (70% HRmax) across all conditions, resulting in a similar perception of effort irrespective of the differences in mechanical load [29].

VO_2_ was significantly lower under hypoxic conditions compared with normoxic conditions. However, VO_2_ in NBRJ showed a tendency to increase with the rise in exercise load, although this difference did not reach statistical significance. Thompson et al. [30] reported that following acute dietary nitrate supplementation, cycling exercise performed at various intensities (50%, 70%, and 90% VO_2_peak) showed that the effect of nitrate supplementation on VO_2_ varied depending on exercise intensity, which may be understood in a context similar to the intensity-dependent VO_2_ response observed in the present study.

Meanwhile, O_2_pulse is an indicator of oxygen uptake per heartbeat, calculated as VO_2_ divided by HR [31]. In the present study, O_2_pulse was significantly higher in NBRJ than in NPLA under normoxic conditions. Given that heart rate was maintained at the target level during exercise, this indicates that oxygen uptake per heartbeat was relatively higher in NBRJ. However, since VO_2_ itself did not differ significantly between NPLA and NBRJ, this finding should be interpreted cautiously as a change in the relative relationship between VO_2_ and HR rather than as an absolute increase in oxygen uptake. In addition, according to the Fick principle, O_2_pulse can also be expressed as the product of SV and the arteriovenous oxygen difference [31]. However, SV did not differ significantly among conditions in the present study. Therefore, it is difficult to conclude that the difference in O_2_pulse resulted from changes in central cardiac function. In this regard, de Castro et al. [32] reported that chronic BRJ supplementation improved VO_2_max, velocity associated with VO2max and peak velocity in 13 male runners, suggesting that BRJ may influence oxygen uptake and exercise performance. However, that study assessed maximal exercise capacity following chronic supplementation, which limits direct comparison with the present study. Meanwhile, no significant difference in O_2_pulse due to BRJ was observed under hypoxic conditions. This may be because, despite the potential facilitation of nitrite-to-NO conversion under hypoxic conditions, the limited oxygen availability reduced the mechanical load and corresponding oxygen demand required to reach the same target heart rate, which may account for the absence of a significant difference in O_2_pulse due to BRJ supplementation under hypoxic conditions.

Regarding VCO_2_, NBRJ was significantly higher than HBRJ but did not differ significantly from NPLA. In addition, no significant difference was observed between HPLA and HBRJ under hypoxic conditions. These results may reflect the combined influence of mechanical load and hypoxia-induced changes in substrate utilization. In NBRJ, the relatively higher mechanical load may have contributed to increased VCO_2_ through elevated metabolic demand. In contrast, in HPLA and HBRJ, despite the lower mechanical load, increased reliance on carbohydrate metabolism under hypoxic conditions may have sustained VCO_2_ levels [11,33]. Regarding RER, HPLA was significantly higher than both NPLA and HBRJ, whereas HBRJ did not differ significantly from NBRJ. The elevated RER observed in HPLA may reflect an increased reliance on carbohydrate metabolism to sustain ATP production in response to the limited oxygen supply under hypoxic conditions, consistent with the mechanism proposed in previous studies [11,33]. The lower RER in HBRJ compared with HPLA, which was similar to that in NBRJ, suggests that BRJ supplementation may have partially attenuated the shift in substrate utilization under hypoxic conditions; however, since the environment × treatment interaction for RER was not significant, this interpretation should be made with caution. Furthermore, as substrate oxidation rate was not directly measured in the present study, caution is also warranted in this regard.

In the present study, Deoxy_Hb was significantly higher in HPLA than in NPLA, NBRJ, and HBRJ. This is likely because the limited oxygen supply under hypoxic conditions increased oxygen extraction in skeletal muscle to meet the same energy demands, resulting in elevated deoxygenated hemoglobin concentration. These findings are consistent with previous studies reporting decreased oxygen delivery to skeletal muscle and increased Deoxy_Hb under hypoxic conditions [11]. Notably, unlike HPLA, HBRJ did not differ significantly from NPLA or NBRJ. Given that BRJ supplementation increases NO bioavailability via the NO_3_^−^–NO_2_^−^–NO pathway [34], this finding may reflect a more effectively maintained oxygen delivery efficiency to skeletal muscle even under hypoxic conditions, partially attenuating excessive oxygen extraction. Specifically, while Deoxy_Hb was significantly elevated in HPLA, HBRJ was statistically indistinguishable from the normoxic conditions (NPLA, NBRJ), suggesting that BRJ supplementation maintained the hypoxia-induced increase in skeletal muscle oxygen extraction at a level close to that observed under normoxic conditions. This is consistent with Bailey et al. [34], who reported that BRJ supplementation reduced muscle fractional O_2_ extraction and attenuated the increase in deoxyhemoglobin during exercise, and is further supported by a previous study reporting that nitrate supplementation restored hypoxia-induced impairments in muscle function and metabolic indices toward normoxic levels under a comparable hypoxic condition (FiO_2_ 14.5%) [35].

Both StO_2_ and Oxy_Hb were significantly lower in HPLA than in NPLA. This is likely because the reduced partial pressure of inspired oxygen under hypoxic conditions decreased arterial oxygen saturation, consequently reducing oxygen supply to skeletal muscle and lowering tissue oxygen saturation, consistent with previous studies [11,36]. In contrast, no significant difference was observed between NBRJ and HBRJ. This raises the possibility that BRJ supplementation may have had some influence on the hypoxia-induced reduction in oxygen saturation; however, since the environment × treatment interaction was not significant, this interpretation should be made with caution. No significant main or interaction effects were observed for Total_Hb. Total_Hb is known to reflect local blood volume in skeletal muscle [37], and the absence of significant differences across conditions suggests that local blood volume was similarly maintained during exercise. Therefore, the changes in skeletal muscle oxygenation observed under hypoxic conditions are interpreted as being primarily attributable to changes in oxygen extraction rather than differences in local blood volume.

In the present study, HR was significantly higher in HPLA than in NPLA. This is likely because the decreased arterial oxygen saturation under hypoxic conditions stimulated peripheral and central chemoreceptors, increasing sympathetic nervous system activity and resulting in a more rapid rise in HR toward the target level during the early phase of exercise. These findings are consistent with previous studies reporting that hypoxic conditions induce a faster HR response than normoxic conditions at the same relative exercise intensity [11,25]. HBRJ did not differ significantly from either NPLA or HPLA.

Taken together, the findings of the present study demonstrated that hypoxic conditions required a lower mechanical load at the same heart rate level while inducing greater physiological stress than normoxic conditions, as evidenced by elevated Deoxy_Hb, reduced StO_2_ and Oxy_Hb, increased RER, and higher HR in HPLA. These results confirm that hypoxic conditions are accompanied by impaired skeletal muscle oxygenation and increased reliance on carbohydrate metabolism due to limited oxygen supply. Among these variables, significant environment × treatment interactions were observed for Deoxy_Hb, HR, and VE. In particular, Deoxy_Hb in HBRJ was statistically indistinguishable from normoxic conditions, indicating a clear attenuation of the hypoxia-induced increase in skeletal muscle oxygen extraction. For RER, although the environment × treatment interaction was not significant, a significant main effect of treatment was observed, and HBRJ did not differ significantly from NBRJ, suggesting a potential influence of BRJ supplementation that warrants cautious interpretation.

This pattern, particularly the recovery observed for Deoxy_Hb, offers a somewhat different perspective from previous studies reporting that BRJ supplementation does not confer a clear benefit on exercise performance under hypoxic conditions [21,38,39]. These studies primarily employed performance-based outcomes such as running or cycling performance and endurance capacity, whereas the present study directly assessed physiological responses during exercise, including metabolic responses, skeletal muscle oxygenation, and cardiac function. This finding, together with the significant environment × treatment interactions observed for HR and VE, suggests that BRJ supplementation may attenuate hypoxia-induced physiological strain toward normoxic levels for select variables, even in the absence of a clear performance benefit; however, this pattern was not consistently observed across all measures.

In summary, BRJ supplementation may serve a complementary role in mitigating hypoxia-induced skeletal muscle deoxygenation, particularly as reflected in Deoxy_Hb. However, as circulating NO_3_^−^/NO_2_^−^ concentrations were not directly measured in the present study, and statistically significant differences between HBRJ and HPLA were not confirmed for most variables, these interpretations should be regarded as preliminary, and future studies incorporating larger sample sizes, direct biochemical measurements, and varying levels of hypoxic intensity are warranted.

## 5. Conclusions

Taken together, the present study demonstrated that hypoxic conditions at the same heart rate level induced greater metabolic stress than normoxic conditions, as evidenced by reduced skeletal muscle oxygenation, increased reliance on carbohydrate metabolism, and elevated cardiovascular strain, despite requiring a lower mechanical load. In contrast, BRJ supplementation partially attenuated this hypoxia-induced metabolic stress. These findings suggest that BRJ supplementation may help bring metabolic stress under hypoxic conditions closer to normoxic levels, providing preliminary evidence for its potential nutritional application across various sports settings. However, since statistically significant differences between the hypoxic BRJ and hypoxic PLA conditions were confirmed only for select variables (e.g., Deoxy_Hb and RER), future studies incorporating larger and more diverse populations, direct measurement of circulating nitrate/nitrite concentrations, varying levels of hypoxic intensity, and different BRJ supplementation protocols are warranted to further elucidate the underlying mechanisms.

## 6. Limitations

The present study has several limitations. First, the sample consisted of 12 healthy adult males with no female participants, and an acute exercise protocol was applied at a single hypoxic intensity (FiO_2_: 14.5%); therefore, caution is warranted in generalizing the findings to other sexes, populations, and hypoxic conditions, and further research on chronic supplementation and varying hypoxic intensities is needed. Second, circulating NO_3_^−^ and NO_2_^−^ concentrations were not directly measured, limiting direct confirmation of enhanced NO bioavailability through BRJ supplementation. Third, the two-way repeated-measures ANOVA with interaction testing minimized unnecessary multiple comparisons within each variable; however, given the relatively large number of outcome variables (14) examined across the study, the cumulative risk of Type I error across variables cannot be entirely excluded. Fourth, a primary outcome was not formally prespecified prior to data collection; therefore, findings for individual variables, particularly those not supported by a significant interaction, should be regarded as exploratory rather than confirmatory. Fifth, although the present study is described as double-blind, blinding was applied only to the supplementation condition (BRJ vs. placebo); the normoxic and hypoxic environments were directly perceptible to participants and could not be blinded. Therefore, the double-blind design should be understood as applying specifically to the supplementation factor rather than to the study as a whole.

## Figures and Tables

**Table 1:** Participant characteristics.

Parameters	Healthy Men (*n* = 12)	Minimum	Maximum
Age (year)	25.1 ± 2.3	21.0	29.0
Height (cm)	179.9 ± 4.4	172.1	185.2
Weight (kg)	83.6 ± 13.3	69.2	110.6
BMI (kg/m^2^)	25.8 ± 3.6	21.1	32.6
Fat-free mass (kg)	67.7 ± 6.1	57.3	77.1
Body fat mass (kg)	16.0 ± 9.2	6.4	33.5
Percent body fat (%)	18.2 ± 7.5	7.7	30.3
70% HRmax (bpm)	136.5 ± 1.5	134.0	139.0

Note. Values are presented as mean ± standard deviation. BMI = body mass index, HRmax = maximal heart rate.

**Table 2:** SpO_2_ during exercise under four experimental conditions.

Parameters	NPLA	NBRJ	HPLA	HBRJ
SpO_2_ (%)	96.93 ± 1.16	97.57 ± 0.45	83.79 ± 2.40	83.36 ± 2.47

Note. Values are presented as mean ± standard deviation. NPLA, normoxic condition + placebo; NBRJ, normoxic condition + beetroot juice; HPLA, hypoxic condition + placebo; HBRJ, hypoxic condition + beetroot juice; SpO_2_, peripheral oxygen saturation.

## Data Availability

The data presented in this study are not publicly available due to privacy and ethical restrictions but are available from the corresponding author upon reasonable request.
